# Entrepreneurship Education and Social Entrepreneurial Intentions: The Mediating Effects of Entrepreneurial Social Network

**DOI:** 10.3389/fpsyg.2022.860273

**Published:** 2022-05-03

**Authors:** H. M. Kamrul Hassan, Barbara Igel, Mohammad Shamsuddoha

**Affiliations:** ^1^Department of Marketing, University of Chittagong, Chattogram, Bangladesh; ^2^Moscow School of Management SKOLKOVO, Moscow, Russia; ^3^AIT School of Management, Asian Institute of Technology, Klong Luang, Thailand; ^4^School of Management and Marketing, Western Illinois University, Macomb, IL, United States

**Keywords:** entrepreneurship education, entrepreneurial social network, social entrepreneurial intention, university students, university

## Abstract

Social entrepreneurship has received considerable recognition from universities in recent years. This study aimed to examine the mediating effect of the entrepreneurial social network on entrepreneurship education and social entrepreneurial intention (SEI) of students at the university level. This study adopted a cross-sectional quantitative approach. A convenience sampling method was utilized to choose 392 students studying at the public and private universities in Chattogram, Bangladesh, who then completed a self-administered survey. The data were then analyzed through partial least squares structural equation modeling (PLS-SEM). Results revealed a significant positive relationship between entrepreneurship education and students’ social entrepreneurial intention on the one hand and between entrepreneurship education and entrepreneurial social network on the other hand. It was also found that entrepreneurial social networks had a significantly positive link with students’ SEIs. Furthermore, the study found that entrepreneurial social networks significantly mediate the relationship between entrepreneurship education and students’ SEI. Based on these outcomes, it is suggested to pay attention to entrepreneurship education further and strengthen the entrepreneurial social network to enhance SEI among students. Research findings have provided valuable insights regarding how entrepreneurship education can significantly impact SEI and emphasize the importance of entrepreneurial social networks as a mediator in social entrepreneurship. This study aims to contribute to the relevant social entrepreneurial literature by providing insights on practical issues related to the role of the entrepreneurial social network at the entrepreneurship education level.

## Introduction

The COVID-19 outbreak has had a severe impact on the global economy, influencing employment, safety, income, education and social support in a way that has never been seen before (McGregor and Pouw, 2017; Hernández-Sánchez et al., 2020). With its rapid and wide-ranging impact, COVID-19 set off unexpected complications to daily life and business operations (Ge et al., 2022). The rapid and devastating spread of the COVID-19 virus has overshadowed any other occurrence, spreading to more and more nations, including established, developing, and rising ones, where the economic effects are as dire (Liñán and Jaén, 2020; Aman et al., 2022). However, due to COVID-19, poverty and a plethora of other social issues are widespread and widely evident, giving various options for social entrepreneurs (Almeida, 2021). As a result, social entrepreneurship emerged as a field of research and gained increasing respect for entrepreneurs’ attempts to increase global social wealth by developing new models for delivering goods and services and offering solutions to social problems, which includes poverty, a lack of education, or provision of health care, that the present economic and social structure seems unable to address (Saebi et al., 2019). Again, the pandemic has a severe and disproportionate impact on young generations, their interest in business, and their psychological wellbeing, particularly at a time when the world economy is contracting as a consequence of resource constraints caused by COVID-19 (Azizi et al., 2021; Li et al., 2021). Moreover, COVID-19 presents a substantial challenge to the education system, including schools and universities, particularly for overseas students and courses, as restrictions on public gatherings with social distance regulations have reduced in-class teaching, resulting in a rapid transition to online teaching techniques using digital networking (Brammer and Clark, 2020; Krishnamurthy, 2020; Maqsood et al., 2021). To address these challenges, there has been rapid adoption of virtual and online learning approaches for entrepreneurship education that incorporate online social networking (Ratten and Jones, 2021; Rauf et al., 2021).

Since worldwide communities grapple with increasing levels of economic, social, and environmental challenges due to COVID-19, studies related to social entrepreneurship have become an increasingly important topic of interest in entrepreneurial literature (Carsrud and Brännback, 2011; Liñán and Fayolle, 2015; Urban, 2020; Margaça et al., 2021). Moreover, the role of social entrepreneurship has been redefined in society due to the enormous impact of COVID-19 on important social issues, such as housing and hunger, and there has been a radical transition among social entrepreneurs from being individual changemakers to community resource organizers (Bacq and Lumpkin, 2021). The number of social enterprises, social enterprise-related programs supported by governments, and, most notably, social entrepreneurial education has been substantially growing (Solomon et al., 2019). The focus of social entrepreneurship is to reform society through resolving social issues regarding social inequality, poverty, unemployment, and environmental degradation, to improve social and economic wellbeing for the emerging world through entrepreneurship (Halberstadt and Spiegler, 2018). Social entrepreneurship is considered a social impact generating practice that can take place within or across voluntary, commercial, or government organizations (Austin et al., 2006). Unlike commercial entrepreneurs, social entrepreneurs, rather than optimizing income, create social values by introducing social changes (Dacin et al., 2010; Estrin et al., 2016). Zahra et al. (2009) defined that social enterprise comprises activities, practices, and procedures to explore and uncover opportunities to strengthen social wealth by establishing new entities or improving existing businesses. Many researchers have acknowledged the significant impact of entrepreneurial networks on an individual’s entrepreneurial intentions, which eventually may enhance firm performance (Walter et al., 2006; Xu and Lu, 2010; Abbas et al., 2019b; Khan et al., 2019). In addition, utilization of entrepreneurial networks along with innovations, knowledge transfer, and social networking enable businesses to increase their revenue and profitability, hence improving their business performance (Liu et al., 2021; Mubeen et al., 2021). The analysis of entrepreneurial social networks has evolved as a significant research theme within the theoretical domain of entrepreneurship (Smith and Lohrke, 2008). Klyver et al. (2008) opined that people networking with entrepreneurs appear more entrepreneurial-centric. Since entrepreneurs’ business specifications and environments are always transforming, entrepreneurial networks, as a method of ensuring their survival, are dynamic and change drastically over time (Soetanto et al., 2018). In social entrepreneurship, the network consisting of an open framework with multiple stakeholders can produce social benefits through bridging gaps and improving the growth of social enterprise (Busch, 2014; Dufays and Huybrechts, 2014; Kokko, 2018). It provides various information and support to the social entrepreneurs needed to develop their organizations, particularly when dealing with challenging situations (Omorede, 2014). Forming a viable social entrepreneurial network answers major social issues and results through major social transformation, which helps continuously improve societies, economies, and the environment. While studying at university, students may acquire entrepreneurial skills through practicing entrepreneurship, backed by education (FE), and through networking with relevant stakeholders (Middleton et al., 2019).

The extensive development of technology and business education in universities has made entrepreneurship a catalyst for the enhancement of the jobs and growth of the modern economy among university students (Trivedi, 2016; Bazan et al., 2020). Considering this, social entrepreneurship has received considerable recognition from universities because of its intense focus on creativity and innovative societal problem-solving skills (Leadbeater, 1997; Martin and Osberg, 2007; Ernst, 2011). Although there is extensive research on entrepreneurial intention, very few research studies have been conducted on SEI (Hockerts, 2015; Liñán and Fayolle, 2015). The current literature available in the area of social entrepreneurial intentions, in particular, is based on studies in Europe and other Western countries, while empirical research in other regions of the world is trivial (Ayob et al., 2013; Tiwari et al., 2017b). Again, relatively very few comprehensive studies have been found on how education systems will develop the attributes in people to become social entrepreneurs focusing on developing countries (Zhang et al., 2014; Arshad et al., 2018; Ndou, 2021). There are only 29 publications on education in social entrepreneurship from 2002 to 2020, compared to 1,500 papers on conventional or general entrepreneurship that have been published since 1988 (García-González and Ramírez-Montoya, 2021). A few formal teaching and learning frameworks exist for social entrepreneurship worldwide (Shahid and Alarifi, 2021). Othman and Ab Wahid (2014) recommended that social entrepreneurship should be discussed, explored, and promoted by education to develop an entrepreneurial environment to empower society. On the other hand, there are very limited studies on how entrepreneurial networks affect social entrepreneurial behavior (Liu et al., 2020). Networking is especially crucial in social entrepreneurship because long-term solutions to large-scale problems with insufficient resources can only be produced by involving various stakeholders and redirecting scarce resources to overlooked social challenges (Dufays and Huybrechts, 2014; Ozeren et al., 2018).

Recently, due to the spread of COVID-19, there has been a full lockdown in different parts of the world, which has even severely affected educational institutions (Zulfiqar et al., 2021). In the wake of COVID-19, educational institutions have been compelled to use online courses to instruct students willingly or involuntary utilizing various learning platforms such as Google Classroom, Microsoft Teams, and Zoom as well as YouTube (McIver and Lepisto, 2017; Alalwan et al., 2019; Zulfiqar et al., 2021). The current COVID-19 dilemma necessitates a shift in entrepreneurship education in light of the digital revolution. Moreover, due to the COVID-19 epidemic, it has been found that people’s desire to become social entrepreneurs has diminished (Ruiz-Rosa et al., 2020). Since society is confronted with a multitude of challenges produced by the COVID-19 crisis that necessitate the development of new knowledge and solutions, entrepreneurship education is critical in the acquisition of information that may aid in the management of the crisis through shaping new ground of research in social entrepreneurship (Ratten and Jones, 2021). Again, because of the emergence of COVID-19, there has been severe damage to contemporary business networks formed through business and professional interrelationships and has offered entrepreneurs the information and competencies necessary for innovation (Zahra, 2021). Networking between students and their local communities has been seriously impacted as a result of COVID-19 affecting extracurricular activities, including sports and theatrical clubs and academic assistance programs such as internships and study trips. However, an online social network platform can re-create typical situations by encouraging students to think and behave in a thoughtful manner and fostering resilience and entrepreneurial mindset in students that are essential in today’s competitive global industry (Ratten and Jones, 2021).

Therefore, the objectives of this study are to identify the role of entrepreneurial networks among university students besides entrepreneurship education in developing SEI. Furthermore, the research findings will demonstrate that entrepreneurship education, entrepreneurial social networking, and SEI are all interrelated, where each has both a direct and an indirect influence on the development of social entrepreneurship in particular, as well as the development of society and the economy in general. By examining this relationship, it will be possible to address that entrepreneurship education institutions should play a critical role in promoting and developing social entrepreneurship by successfully integrating social networking with entrepreneurship education that may generate social transformation and generate employment. This study starts with a basic overview of entrepreneurship education and the formation of entrepreneurial networks. Later, through the development of hypotheses, the concept of social entrepreneurial intention has been studied and its integration with entrepreneurship education and entrepreneurial networks. Following that, the measurements and estimations are explained. Finally, the article discusses limitations, theoretical underpinnings, practical implications, and recommendations and suggestions for further research.

## Literature Review

### Social Entrepreneurial Intention

Intentions encompassing a cognitive focus such as interest, hope, and belief that influence the option of entrepreneurship for an individual is regarded as the most significant indicator of actual behavior (Ajzen, 1991; Peng et al., 2012; Tiwari et al., 2017a). Entrepreneurial intention is characterized as a diligent mindset guided to planned entrepreneurial orientation through personal knowledge and understanding (Do and Dadvari, 2017). Entrepreneurial intention is considered a reliable indicator of entrepreneurial behavior for a person planning to launch a new venture on a long-term basis (Obschonka et al., 2010). A significant surge in Social Entrepreneurial Intention related research, widely regarded as a prominent field of social entrepreneurship studies, has been observed in recent years, and it has become an emerging field of interest for academicians and researchers (Liñán and Fayolle, 2015; Tan et al., 2020). SEI may be termed as cognitive action by persuading individuals to gain information, understand solutions, and undertake social entrepreneurial activities (Mair et al., 2006). To put it another way, SE intentions may be summed up as a desire to start a business or start a social initiative with the goal of doing good for society (Bacq and Alt, 2018). Akhter et al. (2020) opined that students’ intentions toward social entrepreneurship to address underlying societal problems through the establishment of a new start-up would be stimulated by educational assistance provided by a university.

### Entrepreneurship Education

Entrepreneurship education, which plays a critical role in forming entrepreneurial drive and intention among individuals, improves entrepreneurial ability and orientation to encourage entrepreneurial intention among university students (Ferreira and Trusko, 2018). Due to COVID-19, leading to a financial downturn and a significant increase in the unemployment rate considering the recent international crisis, entrepreneurship education has gained a higher exposure, reputation, and possessions than it has ever been (Secundo et al., 2021). Since multiple studies have established a strong correlation between entrepreneurial education and SE, entrepreneurial education has received considerable attention from universities, policymakers, and entrepreneurial researchers (Martín and Cuervo-Arango, 2017; Naveed et al., 2021). In contrast to other scientific fields, entrepreneurship education offers a potential means of guiding individuals on how to cope with the COVID-19 situation (Ratten and Jones, 2021). Moreover, the ongoing pandemic coronavirus has created serious obstacles for academic institutions in teaching and learning and forced them to move to online-based entrepreneurial education by technology adoption to ensure students’ greater degrees of readiness on the contextual and situational conditions, including COVID-19 (Rahmat et al., 2021; Ge et al., 2022; Zhou et al., 2022). Higher education focusing on entrepreneurship requires students to acquire entrepreneurial insights and skills and eventually start a business entity after university degrees. Even though entrepreneurial education plays a crucial role in setting up a new entrepreneurial venture, university support is also vital for entrepreneurship (Saeed et al., 2015; Sidratulmunthah et al., 2018). Earlier studies have indicated that an individual’s education level is associated with their intelligence, competence, confidence, and problem-solving capabilities (Baier-Fuentes et al., 2018). Entrepreneurs with strong business studies backgrounds are more inclined to lead consumer-focused companies (Ganotakis and Love, 2012). The perception and evaluation of students’ entrepreneurial intention need particular importance to design effective academic programs concerning entrepreneurship and business growth (do Paço et al., 2011). Mohamad et al. (2015) suggested that entrepreneurial education, including formal and informal education, should be incorporated in the study curriculum to foster entrepreneurial intentions.

*H1: Entrepreneurship education is significantly related to SEI*

*H2: Entrepreneurship education is significantly related to entrepreneurial social network*

### Entrepreneurial Social Network

Networking is increasingly acknowledged as a crucial element of the entrepreneurial process, and it has emerged as a prominent topic in entrepreneurial studies (Hansen, 1995; Jack et al., 2010). Networks are regarded as a viable source of knowledge, namely the cognitive process of gathering and integrating knowledge, deriving meaningful learning experiences, and producing innovative solutions based on existing knowledge (Soetanto, 2017). An entrepreneurial network is widely acknowledged as being crucial to the entrepreneurial process in the literature on entrepreneurial networks (Chen and He, 2011). Being in an entrepreneurial network is associated with an individual’s capability to start a business efficiently. That is why entrepreneurs must consider the importance of networks in their decision-making process (Elston and Weidinger, 2019). In a number of research findings, entrepreneurial social networking has been identified to have a key positive influence in analyzing entrepreneurial intention (Ng and Rieple, 2014; Ruiz-Palomino and Martínez-Cañas, 2021). Access to entrepreneurial social networks, and hence to essential resources and skills for launching a new firm, may thus serve as a stimulus for progressing through the start-up process and achieving greater success (Wang et al., 2007). Entrepreneurial social networking is gradually receiving exposure in relation to education, with significant implications for changes and adjustments in the teaching and learning field, both specifically for academic as well as research purposes, and initially focused on social relations (Wei et al., 2019). Moreover, in the post-COVID era, the social network is playing an increasingly important role in influencing university students’ learning behavior, which is critical to achieving sustainable education (Abbas et al., 2019a). Student usage of social media and networking technologies at university has grown with a high rate of acceptance; thus, students can better understand social entrepreneurship by using these platforms more broadly (Oprica, 2013; Hamirul Hamizan Roslan et al., 2019). Since students’ perceptions of the usefulness of a network differed, they functioned as a source for evaluating the effectiveness of the entrepreneurial social network.

*H3: Entrepreneurial social network has a direct influence on SEI*

*H4: Entrepreneurial social network mediates the association between entrepreneurship education and SEI*

### Model of the Research Study

**FIGURE 1:**
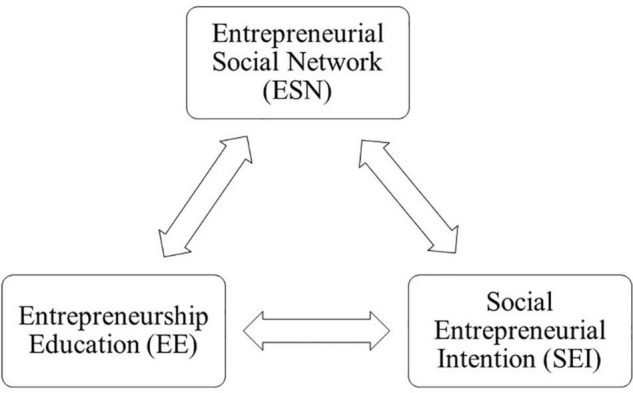
Conceptual framework.

This suggested research model was developed with the primary goal of evaluating the relationship among entrepreneurship education, entrepreneurial social networks, and university students’ intentions toward social entrepreneurship. Three variables have been incorporated into this suggested study’s proposed model. Entrepreneurship education and entrepreneurial social network are the dependent variables in this suggested research model, and this study includes social entrepreneurial intentions as a dependent variable. The structural equation modeling (SEM) approach is suited for this study since the model suggested here incorporates independent and dependent variables (Yan and Yan, 2016; Abbas et al., 2020). Moreover, researchers may readily build up and evaluate hypothetical links between theoretical constructs and those between the constructs and their observable indicators using SEM when there are adequate respondents (Deng et al., 2018). Again, structural equation modeling offers a viable technique for examining associations between latent constructs, particularly important for knowledge-based entrepreneurship (Audretsch et al., 2008). Among the variance-based SEM techniques, partially least squares modeling (PLS) is considered to be the most completely developed and comprehensive system (Henseler, 2017). In comparison to CB-SEM, it is more robust and effortlessly combines both formative and reflecting constructs fitting with both small and large samples (Hair et al., 2011). PLS-SEM, using SmartPLS software can facilitate structural equation modeling solutions with almost any degree of complexities in the structural model and/or constructs (Ringle et al., 2012; Hair et al., 2017). Following a thorough review of the scholarly literature, Figure 1 depicts the conceptual framework for the proposed model.

## Methodology

### Population and Sample

From the literature, it is evident that the majority of internationally acclaimed social enterprises exist in the South Asian region (Reddy et al., 2013; Agrawal and Sahasranamam, 2016). Moreover, research also found that students from emerging nations are more likely to visualize themselves as potential entrepreneurs in the future than those in developed countries (Davey et al., 2011). Therefore, university students, most preferably business students, are usually preferred for evaluation of entrepreneurial intent, considering their representations among individuals engaging in business decision-making and the diversity of entrepreneurial insights, attitudes, and perceptions to this purpose (Krueger and Carsrud, 1993; Lanero et al., 2015; Teixeira and Forte, 2017). Since the entrepreneurial intention is not a clearly measurable factor, a structural equation method is deemed to be adequate (do Paço et al., 2011). Therefore, a convenience sampling technique was used for obtaining a sample of respondents from the public universities in Chattogram, Bangladesh, where entrepreneurship courses have been offered. According to Boomsma and Hoogland (2001), the sample size should be larger than 200 as a standard in most circumstances. Again Reinartz et al. (2009) mentioned that PLS seems to be the preferred approach in all circumstances, where the sample size is less than 250. The 392 respondents’ sample consisted of undergraduate and postgraduate business students for this study.

### Instruments

The current study is carried out through a self-administered questionnaire comprising closed-ended questions. A Likert five-point scale was utilized where the “1” was labeled as “strongly disagree,” and “5” was known as “strongly agree.” Research variables and their measurements are depicted in Table 1.

**TABLE 1:** Measurement items.

Constructs		Item	Source
Entrepreneurship education (EE)	EE1	Entrepreneurship education offers courses related to entrepreneurship.	Adopted from Walter and Dohse (2012) and Ayed (2020)
	EE2	Entrepreneurship education offers management skills focused on social entrepreneurship.	
	EE3	Entrepreneurship education enhances my ability to innovate.	
	EE4	Entrepreneurship education helps me to identify business opportunities.	
Entrepreneurial social network (ESN)	ESN1	Entrepreneurial social network provides me with information and support that may help or encourage me to undertake a new venture.	Adopted from Fernández-Pérez et al. (2015) and Scarmozzino et al. (2017)
	ESN2	Entrepreneurial social network provides online opportunities to discuss new business ideas.	
	ESN3	Entrepreneurial social network provides me with greater access to resources.	
	ESN4	Entrepreneurial social network helps me in meeting lots of people with good ideas for new businesses.	
Social entrepreneurial intention (SEI)	SEI1	I have the skills and capabilities essential to be a social entrepreneur.	Adopted from Ip et al. (2017) and Al-Shammari and Waleed (2018)
	SEI2	I prefer to be an entrepreneur, rather than an employee of an organization.	
	SEI3	My professional goal is to be a social entrepreneur.	
	SEI4	I am determined to create a social entrepreneurial venture in the future.	
	SEI5	I will make all effort to start and run my own social enterprise.	

### Data Analysis

The analysis study discusses two different forms of a hypothesis: direct and mediating. Descriptive statistics has estimated the profile of the respondents. SPSS 23 software is being used for the factor analysis, and inferential statistics is carried out to evaluate the direct and mediating hypotheses using SmartPLS 3.0 software.

## Results and Discussion

The measurement model, also known as the outer model, was utilized to examine the reliability and validity of indicators. In addition, measurement model research was undertaken by analyzing the reflective measurement model, the most widely adopted latent constructs measurement model, where the determination of intention is considered satisfactory applications of the reflective indicator model (Hair et al., 2013).

Descriptive statistics of the participants to explain the sample structure are presented in Table 2. For checking the reliability of latent variables, an assessment of outer loadings is measured. Items with low loadings were removed to maintain the required AVE of 0.5 (Hair et al., 2013; Sarstedt et al., 2017). Composite reliability (CR), Rho_A, and Cronbach’s alpha for assessing internal consistency were found sufficiently high. Rho_A and Cronbach’s alpha is higher than 0.7, implying that the model is reliable and robust (Hair et al., 2013; Sarstedt et al., 2017). Composite reliability ranging from 0.8811 to 0.8891 indicates satisfactory reliability (see Table 3).

**TABLE 2:** Descriptive statistics of respondents.

	Description	Frequency	Per cent
Age	below 20 years	16	0.04
	20–25 years	341	0.87
	above 25 years	35	0.09
Gender	Male	274	0.7
	Female	118	0.3
Education status	Undergraduate level	216	0.55
	Graduate level	176	0.45
Socio-economic status	Lower	5	0.01
	Lower middle	43	0.11
	Middle	279	0.71
	Upper middle	56	0.14
	Upper	9	0.02

**TABLE 3:** Reliability and validity of constructs.

	AVE	Composite reliability	rho_A	Cronbach’s alpha
EE	0.611	0.863	0.793	0.788
ESN	0.662	0.887	0.834	0.830
SEI	0.773	0.911	0.854	0.853

Convergent validity, the next step in assessing the reflective model, was evaluated by Average extracted variance (AVE) and determined from the average of the squared loadings of every single indicator linked to constructs. The optimal level of AVE should be 0.50 or above (Agan et al., 2013; Chekima et al., 2016). The proportion between the square root of AVE and correlations, also known as the Fornell–Larcker criterion, were assessed to measure the discriminant validity shown in Table 4. Findings suggest that the diagonal values (in italics) representing the square root of the AVEs of latent constructs were more significant than their correlations, which indicates that they possess discriminant validity. An additional measure for assessing discriminant validity, the Heterotrait–Monotrait ratio (HTMT), was also examined in this study. Based on consistent loadings, an indicator’s HTMT value is calculated by comparing the average correlations of the indicators across multiple constructs and within each construct (Henseler et al., 2014). The highest value of HTMT in this study was 0.883, which is below the threshold level of 0.90. The results of the HTMT test suggested that the model had considerable reliability and validity.

**TABLE 4:** Fornell-Larcker criterion and Heterotrait-Monotrait Ratio (HTMT).

**Fornell-Larcker criterion**			
	**EE**	**ESN**	**SEI**

EE	0.782	0.000	0.000
ESN	0.661	0.814	0.000
SEI	0.714	0.655	0.879

**Heterotrait-Monotrait Ratio (HTMT)**			

	**EE**	**ESN**	**SEI**

EE	0.000	0.000	0.000
ESN	0.812	0.000	0.000
SEI	0.867	0.775	0.000


Moreover, additional cross-loading scores were higher than 0.7, as shown in Table 5. Loadings greater than 0.70 signify that the construct explains more than 50 per cent of the indicator’s variance, highlighting that the predictor demonstrates an acceptable level of reliability (Chin, 1998; Henseler et al., 2009).

**TABLE 5:** Cross-loadings among measurement scale items.

	EE	ESN	SEI
EE1	*0.730*	0.466	0.479
EE2	*0.782*	0.537	0.567
EE3	*0.800*	0.507	0.566
EE4	*0.813*	0.551	0.612
ESN1	0.496	*0.783*	0.479
ESN2	0.516	*0.845*	0.550
ESN4	0.535	*0.805*	0.503
ESN5	0.595	*0.821*	0.588
SEI1	0.619	0.570	*0.885*
SEI3	0.595	0.591	*0.865*
SEI4	0.668	0.566	*0.887*

*Italic values are loadings for items, which are above the threshold value of 0.7.*

One of the prime requisites for evaluating an inner model that defines the degree of variance explained in every endogenous latent construct is the determinants coefficient (R^2^) (Hair et al., 2012). Cohen (1988) claimed that the value of R is low if it is between 0.02 and 0.12, liberal if it is between 0.13 and 0.25, and above considerable if it is 0.26 and above. In the present study, the R^2^ value suggests that the proposed model describes 56.9% of the overall SEI variance, which is highly significant. The significant variance of the model indicates that both variables can better explain the social entrepreneurs’ intention among students of emerging countries.

This study was conducted to identify the direct effect of entrepreneurship education on the students’ SEIs before introducing the mediator variable. As shown in Table 6, the outputs of the model showed a significant relationship between entrepreneurship education and students’ SEIs, β = 0.500, *T* = 10.557, *p* < 0.01, which supports to accept H1, indicating that entrepreneurship education determines the SEIs of students and suggests that an improvement in entrepreneurship education will correspondingly increase student SEIs. In fact, it means that entrepreneurship education increases the willingness of the student to pursue a social entrepreneurial career. Results are consistent with the findings of earlier studies investigating the association between entrepreneurship education and entrepreneurial intention and suggest that entrepreneurship education can significantly impact the intentions of students to launch a new business (Hockerts, 2018; Naveed et al., 2021).

**TABLE 6:** Path coefficients.

**Direct effects**						
**Hypothesis**	**Relationship coefficient**	**Std beta (β)**	**Standard error**	**T statistics**	***p*-value**	**Decision**

H1	EE – >ESN	0.661	0.043	15.523	0.000	Supported
H2	EE – >SEI	0.500	0.047	10.557	0.000	Supported
H3	ESN – >SEI	0.324	0.056	5.746	0.000	Supported

**Indirect effect**						

**Hypothesis**	**Relationship coefficient**	**Std beta (β)**	**Standard error**	**T statistics**	***p*-value**	**Decision**

H4	EE – >ESN – >SEI	0.214	0.041	5.268	0.000	Supported

**FIGURE 2:**
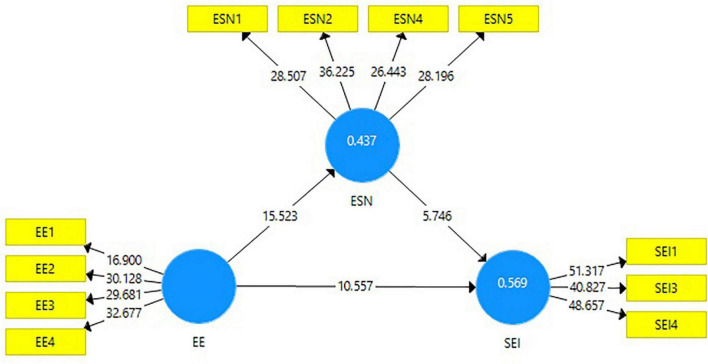
The structural model.

Secondly, our model (see Figure 2) showed a positive association between entrepreneurship education and entrepreneurial social networks, β = 0.661, *T* = 15.523, *p* < 0.01, which supports accepting H2. Our findings are consistent with the results of previous studies that examined the links between entrepreneurship education and social network. Earlier literature also suggested that social networks facilitate learning opportunities to enhance entrepreneurship education (Erjavec, 2013; Yang et al., 2014; Greenhow and Askari, 2017). Consequently, the current model showed a significant relationship between the entrepreneurial social network and students’ SEIs, with β = 0.324 and *T* = 5.746, *p* < 0.01, which supported accepting H3, indicating that the growth of a student’s social network improves the student’s SEIs by increasing the student’s interest in a social entrepreneurial career. Again, previous research on the interaction between the entrepreneurial social network and entrepreneurial intention found similar results (Hussain and Norashidah, 2015; Ratten et al., 2016).

### Mediation Effect

The current study has analyzed a simple mediation approach where the impact of entrepreneurship education on SEI might well be mediated by mediating variable entrepreneurial social networks. According to Baron and Kenny (1986), a mediating effect is obtained if the independent and mediator variable is constrained, and the previously pertinent relationship of the independent variable with the dependent variable found significant will significantly diminish. After estimating the effect of mediation of entrepreneurial social networks on the relationship between entrepreneurship education and students’ SEIs through the bootstrapping approach, it is estimated that entrepreneurial social network mediates the relationship between entrepreneurship education and SEI. Initially, entrepreneurship education affects SEIs, and it is statistically significant at 0.01 level (Figure 2); when the entrepreneurial social network is introduced as a mediator, the coefficient value and its *T*-value decrease (β = 0.214, *T* = 5.268), although it remains significant. This indicates that the relationship between entrepreneurship education and students’ SEIs, which was statistically significant before, changed significantly with the inclusion of entrepreneurial social networks in the model. It means that there is a mediation effect of entrepreneurial social networks in explaining the relationship between entrepreneurship education and SEI, and hence from the findings, H4 is supported. According to Nitzl et al. (2016), if both the direct and indirect effects are proven to be significant, it is considered partial mediation. That is to say, entrepreneurial social network partially mediates the association between entrepreneurship education and SEIs concerning university students (Table 6).

## Conclusion

### Theoretical Contribution

Earlier studies predicting SEI incorporated social networking with personality traits (Nga and Shamuganathan, 2010) and perceived supports (Tran and Von Korflesch, 2016; Younis et al., 2020). Again, identifying the role of education in SEI, Shahverdi et al. (2018) recognize obstacles to SEI by moderating the educational role, and Hockerts (2018), in effect, focuses on the link between the experience-based learning process and the movement toward social entrepreneurship. Therefore, it is evident that entrepreneurship education is basically concerned with generating innovative skills and knowledge, albeit less intention was paid to the social network (Nielsen and Gartner, 2017). Moreover, no research highlighted the importance of entrepreneurial social networking in entrepreneurship education while studying SEI. Moreover, entrepreneurial social networking has also not been considered in earlier studies as a mediating variable in developing SEI. The noteworthy finding of this study is the significant role of entrepreneurial social networking in students’ entrepreneurship education while developing SEI; therefore, more comprehensive research is required in social entrepreneurship that incorporates entrepreneurial social networking as a critical factor in developing social entrepreneurs in higher education institutions. Schøtt (2017) argued that platforms for enhancing entrepreneurial intentions need education as human capital and social networking as social capital. In this regard, this study makes a significant contribution to theory by investigating the entrepreneurship education system and acknowledging entrepreneurial social networking as a critical mediating variable that significantly impacts SEI, thereby generating economic rent in the society. This study also confirms that entrepreneurship education, entrepreneurial social networking, and SEI are truly interdependent and that these have a direct and an indirect impact on social entrepreneurship development in particular and the society and economy in general. Our findings add value to the social entrepreneurship theory and encourage research exploring the interaction between entrepreneurship education and entrepreneurial social networking as a catalyst for social entrepreneurship development in different emerging and mature economies.

### Managerial Contributions

This research undoubtedly has strategic importance from an entrepreneurial and managerial perspective. Without awareness of the strategic implication of social networking, it is almost impossible for a social entrepreneur to formulate and implement strategies in a proactive manner for the growth and development of the enterprise. After gaining knowledge about critical issues that drive social entrepreneurship, social entrepreneurs can take positive steps to acquire, control, and allocate the economic resources at the right time in the right place for the right purpose to achieve predetermined social entrepreneurial goals followed by missions and visions and thereby fulfill the often-incompatible expectations of various stakeholders. And undoubtedly, the university will work as the pollination ground for young entrepreneurs where they will be guided and inspired as future social entrepreneurs. The interest in social entrepreneurship is continuously growing among young students because of its promising contribution to addressing pressing challenges and the socio-economic consequences for humankind. Given that SEI depends on education, university students can get a direct orientation toward SEI if an adequate infrastructure for the entrepreneurship education system is ensured, which seems impossible without an entrepreneurship oriented social network. Moreover, emerging economies facing numerous socio-cultural challenges, like Bangladesh, can utilize entrepreneurial social networking in the entrepreneurship education system to increase SEI motivation by providing experiential learning opportunities alongside formal university education.

This research adds important insights into the contribution of social networking in entrepreneurship education institutions that are useful for developing effective education policies. This study shows that academic institutions focused on entrepreneurship education play a critical role in promoting and developing social entrepreneurship by successfully integrating social networking with entrepreneurship education, which might encourage social transformation and generate job opportunities. This study also suggests that university authorities should offer students entrepreneurship education programs and networking with recognized entrepreneurs who can provide critical experiential learning opportunities from practice in the real world.

Recently, the outbreak of the pandemic COVID-19 has triggered a significant crisis in the overall education sector. Hence, the current crisis provides an opportunity for academicians to consider and encourage possible alternative solutions through forming communities and sharing experiences in entrepreneurial social networks that can help students develop intention toward social entrepreneurship to deal with the crisis. This study implies that integrating entrepreneurial social networks with entrepreneurship education can offer active learning assistance and more incredible opportunity to overcome challenges. Therefore, academic staff needs to be better prepared by providing appropriate training to build student support systems in a crisis that can encourage new positive participation and social resilience, which benefit students and their education, university stakeholders, and society.

### Limitation and Further Research

As the current exploratory study method applied PLS-SEM and convenience sampling with limited respondents, future researchers should re-examine our findings from different perspectives and model the proposed relationships with larger samples selected by probability-based sampling and a covariance-based SEM. In order to do this, researchers can extend the size of the data considering other urban and rural locations and contexts in Bangladesh and the South Asian region. As the research was undertaken in Bangladesh, comparative analysis in other emerging economies could test the research design presented here to better understand the sources of entrepreneurial intentions in other South Asian and South-East Asian countries with some shared cultural context and socio-economic similarity exists. Since the current research is based on a cross-sectional study and assumes that the entrepreneurial action results are limited, a longitudinal study can add new insights by comparing short, medium- and long-term development patterns of SEI over the span of several years moving through the education system. Moreover, a generalization of our results may be limited by the context of our data collection being limited to Chattogram, Bangladesh.

According to the findings of this study, the mediating effect of entrepreneurial social networks between entrepreneurship education and SEI in the contexts of Bangladesh was critically analyzed, taking into account both public and private university business students. In line with the previous study undertaken in geographically and culturally disparate nations, the findings point in the direction of additional investigation into the interrelationship between entrepreneurship education, entrepreneurial social networks, and the SEI. As a result, a comprehensive evaluation is necessary to ensure that each factor works in harmony to produce value and achieve predetermined social entrepreneurial goals for socio-economic progress.

## Data Availability Statement

The raw data supporting the conclusions of this article will be made available by the authors, without undue reservation.

## Ethics Statement

Ethical review and approval was not required for the study on human participants in accordance with the Local Legislation and Institutional Requirements. The patients/participants provided their written informed consent to participate in this study.

## Author Contributions

HH conceived the idea, designed the general framework of the study, and wrote a preliminary version of the manuscript. BI participated in the design of the analyses of the findings, the discussion of the results and conclusions. MS provided critical feedback and helped shape the manuscript. All authors contributed in the review and editing of the final manuscript.

## Conflict of Interest

The authors declare that the research was conducted in the absence of any commercial or financial relationships that could be construed as a potential conflict of interest.

## Publisher’s Note

All claims expressed in this article are solely those of the authors and do not necessarily represent those of their affiliated organizations, or those of the publisher, the editors and the reviewers. Any product that may be evaluated in this article, or claim that may be made by its manufacturer, is not guaranteed or endorsed by the publisher.
